# iVirus 2.0: Cyberinfrastructure-supported tools and data to power DNA virus ecology

**DOI:** 10.1038/s43705-021-00083-3

**Published:** 2021-12-14

**Authors:** Benjamin Bolduc, Olivier Zablocki, Jiarong Guo, Ahmed A. Zayed, Dean Vik, Paramvir Dehal, Elisha M. Wood-Charlson, Adam Arkin, Nirav Merchant, Jennifer Pett-Ridge, Simon Roux, Matthew Vaughn, Matthew B. Sullivan

**Affiliations:** 1 Department of Microbiology, The Ohio State University, Columbus, OH USA; 2 Center of Microbiome Science, Columbus, OH USA; 3 EMERGE Biology Integration Institute, Columbus, OH USA; 4 Environmental Genomics and Systems Biology Division, E.O. Lawrence Berkeley National Laboratory, Berkeley, CA USA; 5 Department of Bioengineering, University of California, Berkeley, CA USA; 6 The University of Arizona, Tucson, AZ USA; 7 Physical and Life Sciences Directorate, Lawrence Livermore National Laboratory, Livermore, CA USA; 8 Life & Environmental Sciences Department, University of California Merced, Merced, CA 95343 USA; 9 DOE Joint Genome Institute, Lawrence Berkeley National Laboratory, Berkeley, CA USA; 10 Texas Advanced Computing Center, The University of Texas at Austin, Austin, TX USA; 11 Department of Civil, Environmental and Geodetic Engineering, The Ohio State University, Columbus, OH USA

**Keywords:** Biological techniques, Ecology

## Abstract

Microbes drive myriad ecosystem processes, but under strong influence from viruses. Because studying viruses in complex systems requires different tools than those for microbes, they remain underexplored. To combat this, we previously aggregated double-stranded DNA (dsDNA) virus analysis capabilities and resources into ‘iVirus’ on the CyVerse collaborative cyberinfrastructure. Here we substantially expand iVirus’s functionality and accessibility, to iVirus 2.0, as follows. First, core iVirus apps were integrated into the Department of Energy’s Systems Biology KnowledgeBase (KBase) to provide an additional analytical platform. Second, at CyVerse, 20 software tools (apps) were upgraded or added as new tools and capabilities. Third, nearly 20-fold more sequence reads were aggregated to capture new data and environments. Finally, documentation, as “live” protocols, was updated to maximize user interaction with and contribution to infrastructure development. Together, iVirus 2.0 serves as a uniquely central and accessible analytical platform for studying how viruses, particularly dsDNA viruses, impact diverse microbial ecosystems.

Microbiome researchers are revealing the power of microbes that live in, on, and around us to shape human health and Earth’s diverse ecosystems [1, 2]. These advances have been aided by myriad analytical capabilities and platforms that help researchers better “see” microbes using gene marker and metagenomic sequencing data (see Supp. Table 1). Though there is much to learn, it is increasingly clear that viruses modulate these microbial impacts. For example, in the oceans, every day viruses lyse one in three cells, transfer 10^29 genes from one host to another [3–5], and alter global biogeochemical cycles through lysis products, virus-encoded auxiliary metabolic genes that impact photosynthesis [6, 7], carbon [8, 9]/nitrogen [10, 11]/sulfur cycling, and metabolic reprogramming [12]. Similar anecdotes of virus ecosystem impacts are emerging in soils where viruses infect key carbon cyclers and encode genes that modulate carbon cycling [13, 14], and extreme environments where viruses can encode genes that alter their microbial host’s abilities to survive such stressful environments [15–18]. As a field, we are on the cusp of a great leap forward in understanding viral roles across diverse ecosystems, with conceptual formulations already emerging [19, 20], and large-scale datasets emerging that are ripe for deep virus-focused exploration [13, 21–24].

Problematically, two key bottlenecks currently prevent researchers wanting to more broadly understand virus impacts in their ecosystem of choice: a) the toolkit for viruses is different to that of microbes, and b) even if a virus toolkit exists, it may not be as mature as for other ecosystems [25]. The main challenges are that viruses lack hallmark genes, have different and more complex taxonomies, and are less well represented in databases. Fortunately, at least for dsDNA viruses, these issues are being resolved as follows: (i) quantitative metagenomic approaches are now available [26, 27], (ii) community consensus [28, 29] and tools [30–33] are emerging for genome-based taxonomy, and (iii) larger datasets with improved analysis methods techniques are expanding coverage of sequence space [29]. Given the importance of viruses and the rapid and growing advances in the emergent field of “virus ecogenomics”, users need access to virus-specific analytical tools, data, and cyberinfrastructure capabilities.

To this end, we developed iVirus as a simplified, user-friendly, publicly accessible resource that is linked to “living” documentation allowing for community feedback and consensus-building [34]. iVirus was originally launched as a limited set of virus ecology-specific apps and datasets deployed on the CyVerse cyberinfrastructure (formerly the iPlant Collaborative) to provide free access to computing, data management, storage, and analysis toolkits [35]. iVirus developers built custom apps and adapted publicly available tools for use in CyVerse along with depositing diverse virus datasets into the CyVerse Data Store. Alongside CyVerse resources, iVirus made several protocols available on the “live protocols for the life sciences” protocols.io website [36] with screenshots, notes, and insights for step-by-step use of each app. This *version 1* iVirus effort offered 7 apps, numerous data projects (75 viromes and 121 virus genomes totaling 1 Tb of data), and 5 “live” protocols. Together this iVirus implementation provided researchers—from beginners to experts—the ability to process virus sequencing datasets from raw reads to taxonomically-classified virus genomes, and establish baseline ecological insights. Further, researchers have had unique and varied opportunity to grow and mature the field through feedback venues including (i) the “live protocols”, (ii) international viromics training workshops (https://u.osu.edu/viruslab/viromics-workshop/), and (iii) VERVE-net community networking efforts [37]. Since its launch, iVirus has grown into a unique and valuable community resource as a ‘top 10’ bioinformatic protocol at protocols.io (~30 K unique views) and thousands of users running iVirus apps at CyVerse. Though other online platforms offer partially overlapping capabilities, these tend to focus specifically on phage genomics (e.g., PHASTER [38], PhageWeb [39], and phage.ai [40]) or were pioneering efforts in this space that are currently under-supported or discontinued (e.g., VIROME [41], Metavir [42]). Thus, iVirus stands as the most comprehensive analytical option for virus ecogenomics.

Not surprisingly, virus ecogenomics has advanced rapidly since iVirus’s introduction in 2017. Beyond new analytical capabilities and data resources, there are also additional cyberinfrastructures of relevance, in particular the Department of Energy’s (DOE) Systems Biology KnowledgeBase (“KBase”) whose goal is to “meet the grand challenge of systems biology—predicting and designing biological function on a range of scales, from the biomolecular to ecological” [43]. While KBase is powerful in the microbiome space, with over 160 apps, until 2019, it lacked any specific for viruses, which limits KBase users studying viruses to non-specialized tools and/or shuffling data between KBase and other tools.

Here we present iVirus 2.0, which offers the first integration of virus ecogenomics tools in KBase, as well as upgrading and expanding CyVerse capabilities with 20 new and updated apps, datasets, and protocols. We continue to provide extensive documentation and seek community feedback, as this motivates iVirus improvements, and helps us assist virus-interested researchers.

## Towards diversifying cyberinfrastructures to broaden community capabilities and access

We first implemented several core iVirus apps on KBase [43] to empower KBase researchers with virus analytics. Both the KBase and CyVerse cyberinfrastructure platforms share some features, including the following: (i) free to use, (ii) hundreds of apps within user-friendly interfaces, (iii) mechanisms for reproducible informatic research, and (iv) committed funding. However, there are differentiating features that might drive researchers towards one platform or another depending upon preferred modes of interaction (summarized in Table 1). Briefly, CyVerse takes a traditional operating system approach with apps, data, and analyses treated as separate entities and currently has more virus analytics available, whereas KBase is designed to encapsulate entire methodological pipelines (apps, data and research notes) into notebooks to be shared publicly or with collaborators, akin to a digitized research paper methods section and which currently include a basic virus workflow (specifics below). From here, we seek to walk readers through virus ecogenomic analyses and the upgraded and new capabilities available on these platforms.

**Table 1:** Broad comparison between CyVerse and KBase Cyberinfrastructures.

	Pros	Cons
CyVerse	• Large number and wide selection of available apps	• Difficult to identify app function and only Agave-based apps are organized
	• File storage “structure” is similar to most user operating systems	• Documentation is scattered and exists at several locations
	• Access to large-scale HPC resources at TACC, access to huge-memory (>1 TB) nodes	• App documentation is often limited, relying heavily on developer good will
	• Low developer cost to app development	• Analyses cannot be shared, though their underlying data can
	• Datasets can be assigned DOIs	
KBase	• Narratives provide clear experimental history	• Limited ability to intuitively search, organize, and browse through data
	• Intuitive app organization	• Limited app selection for various analysis steps
	• User-friendly sharing capabilities of Narratives	• High developer cost to app development
	• Robust Software Development Kit (SDK) for developers	• Limited resources are bottleneck for heavy memory-using apps
	• Narratives can be assigned DOIs	

To provide an overview of the two cyberinfrastructures used for iVirus, we have collated user-informed lists of pros and cons of each. Depending on user experience and skillset, several pros and cons may be reversed.

## iVirus app developments

When a researcher has new microbiome sequence data, whether virus-targeted or microbial, much of the front-end processing is identical. These include read quality control, assembly, gene calling and annotation, sequence alignments, and diverse file manipulations including compression, splitting paired-end reads, and converting between various file formats. As of March 2021, more than 1020 CyVerse and 220 KBase apps, respectively, can be leveraged with these goals in mind (see http://tinyurl.com/4ndkt4n2 and https://kbase.us/applist/, respectively). For iVirus development, we sought to complement these with virus-specific apps and resources to maximize virus inference in complex communities, with a focus to date on dsDNA viruses.

Initially, as implemented in 2017, iVirus created a minimal working pipeline of seven apps that allowed researchers to process a virus metagenome from raw sequencing reads to assembly and conduct analyses via identification, classification, and ecology measured at the level of genes [34]. Specifically, these seven original apps allowed researchers to identify viruses (VirSorter [44]), taxonomically classify them (vConTACT [45]), estimate their abundances (BowtieBatch/Read2RefMapper [34]), cluster open reading frames into protein clusters (PCPipe [34]), and perform read-based community comparisons (Fizkin [46]). In iVirus 2.0, 12 apps have been added to the original 7 (see Table 2), more than doubling the number of apps available to a total of 19 apps. Beyond expanding analytical capabilities—including archaeal and RNA virus identification, genome annotation, virus AMG curation and host prediction— these additional apps also provide choice(s) at each “stage” of the virus ecogenomic pipeline. Below, we describe the newly added and/or updated apps on CyVerse, as well as provide an overview of virus pipeline capabilities currently available for each of the CyVerse and KBase cyberinfrastructures.

**Table 2:** iVirus-powered apps on KBase and CyVerse.

App name	Virus ecogenomics processing stage	Availability	iVirus Version	Reference
vConTACT^a^	Virus Classification	CyVerse	1, 2	Bolduc [45]
vConTACT-PCs	Virus Classification	CyVerse	1, 2	Bolduc [34]
vConTACT2	Virus Classification	CyVerse/KBase	2	Jang [30]
BowtieBatch	Read mapping	CyVerse	1, 2	Bolduc [34]
Read2RefMapper	Read mapping	CyVerse	1, 2	Bolduc et al. [45]
VirSorter	Virus Identification	CyVerse/KBase	1, 2	Roux [44]
VirSorter 2	Virus Identification	CyVerse	2	Guo [47]
PCPipe	Protein clustering	CyVerse	1, 2	Hurwitz [46]
Fizkin	Community profiling	CyVerse	1, 2	Hurwitz [46]
CheckV	Virus analysis	CyVerse	2	Nayfach [60]
MArVD	Virus identification	CyVerse	2	Vik [53]
MArVD2	Virus identification	In development	2	Vik [54]
Cenote-Taker2	Annotation	CyVerse	2	Tisza [55]
MetaPop	Population genetics	CyVerse	2	Gregory [22]
VirMatcher	Virus-Host prediction	KBase	2	Gregory [63]
DRAM-v	AMG identification and annotation	KBase	2	Shaffer [57]
WIsH	Virus-Host prediction	CyVerse	2	Galiez [59]
DeepVirFinder	Virus Identification	CyVerse	2	Ren [48]
VIBRANT^b^	Virus Identification	CyVerse	2	Kieft [51]
MARVEL	Virus Identification	CyVerse	2	Amgarten [49]

^a^vConTACT is comprised of several apps, including *-Gene2Genome, and *-prePCs.

^b^Not integrated by the iVirus team, but included due to its relevance for virus ecogenomics.

### CyVerse

**Fig. 1:**
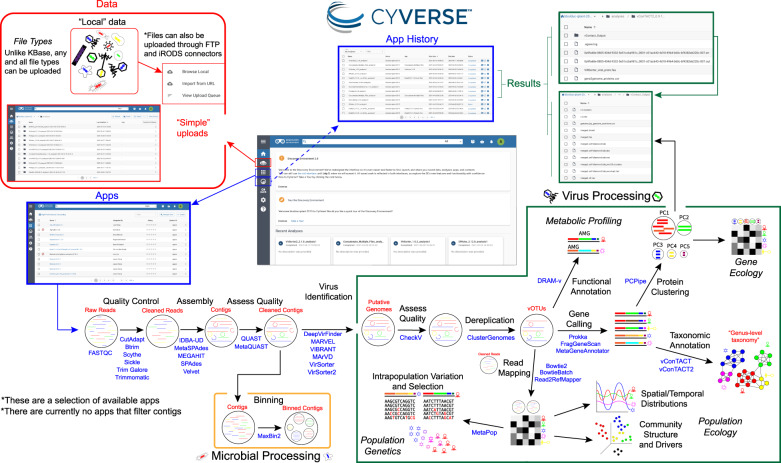
Overview of the iVirus ecosystem of apps and workflow available on the CyVerse Discovery Environment. Within this CyVerse environment, users will upload and process their data (red) through the iVirus apps (blue), and output in a result directory (green). The middle window depicts the main dashboard, from where users can select which task they want to perform (e.g., check a job, access results directory).

Figure 1 provides an overview of the updated “iVirus at CyVerse” pipeline, including relevant aspects of the CyVerse Discovery Environment and virus and microbial tools available.

#### Virus identification (updated)

The first step that is unique to viromics is to take quality-controlled and assembled contigs and identify those contigs that are viral. Originally, we provided VirSorter for this task, which was one of the first virus identification tools to handle fragmented genomes and sequences not closely associated with virus reference sequences [44]. As a major upgrade, we have now added VirSorter2 [47] to iVirus, which uses more genomic features, and applies machine learning to identify virus sequences with improved accuracy along with multiple classifiers (machine learning models) to extend its identification outside of dsDNA phages to include giant viruses (i.e., viruses belong to Nucleocytoviricota, also known as nucleocytoplasmic large DNA viruses (‘NCLDVs’), virophages, ssDNA and RNA viruses.

#### Virus identification (new)

To offer users more choices in this critical first step in the virus ecogenomics workflow, we added several new apps. First, DeepVirFinder (DVF), a deep-learning based virus identification tool, which was the first such tool to employ deep learning [48]. Unlike the features used in VirSorter 1 and 2 and in MARVEL [49], DVF relies on features that allow predictions for contigs of lengths as small as 300 bp, and is overall the superior method for identifying smaller virus contigs [50] (e.g., 3-kb or lower). Second, we added MARVEL to CyVerse. Conceptually, MARVEL [49] is another virus identification tool that uses genomic features to identify viruses and, like VirSorter2, MARVEL uses a machine-learning classifier. Benchmarks show similar specificity and improved sensitivity against VirSorter1, but lower than VIBRANT and VirSorter2 [47]. Though MARVEL requires individual fasta files for each genome, we adapted the MARVEL CyVerse app for scalability by allowing a concatenated input file that our app splits, processes, and then concatenates the separated outputs (with temporary file clean-up). Third, VIBRANT [51] is now integrated. VIBRANT, which stands for Virus Identification By iteRative ANnoTation, uses neural networks with HMMs from a variety of databases (e.g., KEGG, Pfam, VOG), along with a “v-score” to identify a diverse range of viruses, including dsDNA, ssDNA and RNA viruses. For sequences 1 kb or larger and with at least four genes, benchmarking [50] showed VIBRANT out-performing VirSorter 1, VirFinder and MARVEL, while being comparable to VirSorter2. Additionally, VIBRANT also characterizes metabolic pathways to identify virus-encoded auxiliary metabolic genes (AMGs, see below). While VIBRANT was integrated by external researchers outside our iVirus team, it is described here because of its relevance to the field as an additional available virus identification tool.

#### Archaeal virus identification (new)

Because most genome-sequenced archaeal virus isolates derive from extreme environments, these viruses remain difficult to identify from “normal” environments—even when archaea are abundant (e.g., the deep ocean [52]). To help with identification of mesophilic archaea viruses, MArVD, the Metagenomic Archaeal Virus Detector [53], and its most recent machine-learning-powered version, MArVD2 [54], are available in CyVerse.

#### Virus genome annotation (new)

Once virus contigs have been identified, researchers seek to annotate them to understand identifiable functional capacity. In the original iVirus release, no such tools were available beyond standard microbial tools that predicted open reading frames and searched databases one at a time. Since that time, however, two options have emerged with specific value for viruses – Cenote-Taker [55, 56] and DRAM-v [57].

The first, Cenote-Taker [55], was designed to primarily identify and characterize circular DNA viruses (primarily focused on ssDNA virus discovery from animal samples), through the detection of circular sequences via direct terminal repeats (DTR), overlapping ends, and comparisons to known viruses in public databases. It also provided extensive processing of ORFs and output annotated genomes in GenBank-compliant files. The second version of Cenote-Taker [56] added flexibility to discover and annotate all virus classes with DNA or RNA genomes (via hallmark gene models), genome annotation maps, and prophage detection—though performance benchmarks are not available. Because Cenote-Taker has extensive database and software dependencies, local installs are challenging and costly to maintain, which makes it ideal on a cyberinfrastructure like CyVerse.

The second tool, DRAM, represents a different annotation strategy [57]. Specifically, DRAM offers a scalable means to annotate metagenome-assembled genomes, or MAGs, from a pathways perspective that distills resultant gene lists into pathways to better resolve metabolic context. Additionally, a suite of scoring and flagging features comprise a virus-specific portion of this tool, DRAM-v. These help semi-automate identification of virus-encoded AMGs and, critically, curate against functionally interesting metabolic genes that are cellular- rather than virus- encoded (conceptual guidelines and recommendations for this are also available [50]). To promote inter-operability, VirSorter2 offers compatible output for DRAM.

Beyond these two tools, as mentioned above, VIBRANT also includes AMG annotation capabilities. Specifically, VIBRANT uses KEGG annotations to identify novel functions in the new viruses it identifies, though no flagging features are implemented to evaluate certainty around the putative AMG being virus-encoded.

#### Virus-Host identification (new)

Accurately predicting the host(s) of an uncultivated virus using only genome information remains a major challenge with several approaches currently used to link viruses to their hosts including tRNAs, the presence of prophage genes, shared genes between viruses and hosts (e.g., AMGs), CRISPR spacer matches, and k-mer based signatures [58]. Among these, the latter method of using k-mer similarity comparisons between virus and host genomes, has been automated via a tool called WIsH [59] (Who Is the Host), which we have now integrated into CyVerse. Another host prediction tool incorporating WIsH, VirMatcher, is available as a KBase app (see below).

#### Virus genome quality control (new)

A long-standing problem in virus ecogenomics has been how to determine completeness and purity of newly discovered viruses. For this task, CheckV [60] was recently developed and integrated into CyVerse. Briefly, CheckV assesses single-contig virus genome quality, including identification of host contamination for integrated proviruses, estimating completeness for genome fragments, and identifying closed genomes, and then summarizes this for each genome using the community established MIUViG (Minimum Information about an Uncultivated Virus Genome) quality standards [29]. Though CheckV is a major step forward, estimating the completeness of divergent and/or novel viruses remains challenging due to the requirement for “closely related” reference genome sequences.

#### Virus clustering and classification (updated)

Once new viruses are discovered, they need to be taxonomically classified, which is a major challenge for viruses since they lack any universal gene markers. Fortunately, a population genetic grounded biological species definition of 95% average nucleotide identity across shared genes and 85% coverage along the shorted contig was established and confirmed for several marine viruses [21, 61], and now largely adopted as virus operational taxonomic units or “vOTUs” by the virus ecogenomics community [29]. In iVirus, users can dereplicate observed virus genomes in their data into vOTUs using the clustering tool ClusterGenomes (https://bitbucket.org/MAVERICLab/stampede-clustergenomes). Complementarily, higher-level taxonomy can be resolved using gene-sharing network approaches that result in virus clusters (or VCs) that are remarkably concordant with the genera defined by the International Committee on the Taxonomy of Viruses. As such, unknown viruses can be classified with a relative confidence close to ICTV accuracy, at scale. The tool for this, vConTACT [45], was implemented in the original iVirus, and has now has been upgraded to vConTACT2 [30], which offers a new clustering algorithm, confidence metrics, and improved scalability.

#### Population genetics (new)

As sequencing and assembly algorithms have advanced, virus ecogenomics researchers are now able to advance from gene- to population-based studies [62]. With this advance, there is a need to understand variation both between (macro-diversity) and within (micro-diversity) populations. To this end, MetaPop was integrated into iVirus as it offers a simplified analytical and visualization pipeline for population-based inferences in both microbial and virus communities [63]. Specific MetaPop outputs include alpha- and beta-diversity metrics for macrodiversity, as well as microdiversity metrics including the identification of single nucleotide polymorphisms (SNPs; notably including codon-constrained linkages of SNPs), nucleotide diversity (pi and theta), selective pressure (pN/pS, Tajima’s D), and genomic differentiation (Fst) between populations.

### KBase

**Fig. 2:**
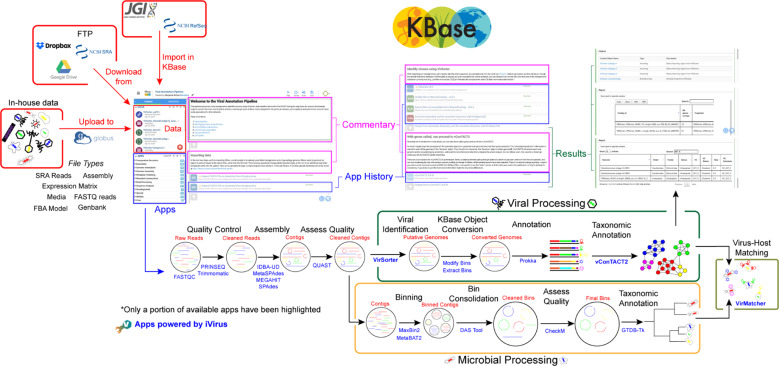
Overview of the iVirus ecosystem of apps and workflow available on KBase. The left window depicts the main dashboard, broadly divided into “Data” (red), “Commentary” (pink), “Apps” (blue), and “Results“ (green).

To increase the accessibility of virus ecogenomics analyses, we have added basic virus ecogenomic functionality to the KBase platform (overview in Fig. 2). We chose to duplicate the core iVirus apps from CyVerse to KBase due to the complementarity of the two platforms, with KBase in particular including a unique interface, distinct user base, and unique microbiome and experimental tracking capabilities (Table 1). KBase also includes a unique virus–host prediction tool, VirMatcher.

#### Virus identification (new)

Currently only VirSorter 1, described above and which performs well for dsDNA phages, has been integrated into KBase.

#### Virus-Host identification (new)

As mentioned above, in silico virus-host predictions remain challenging in virus discovery, and each available tool tends to rely on distinct genomic features for its predictions. Often, however, host prediction results between tools can differ for the same virus sequence, and cross-validation among thousands of predictions is currently a manual process. Thus, we developed VirMatcher, a taxon-aware virus-host predictor that aggregates results from all these methods and uses previous benchmarking experiments to assign a best host prediction and provide a confidence score for each prediction. While the tool is unpublished, its methodology was used to predict nearly 50% of hosts from 33,242 virus genomes for the human gut virome database [22].

## Beyond the apps—iVirus data resources and documentation

Beyond keeping pace with updated and novel capabilities in virus ecogenomics, recent iVirus upgrades include growing the underlying datasets available. Currently, large databases of virus genomes extracted from metagenomes such as IMG/VR [23] have been collected from large-scale mining of publicly available (mostly) microbial metagenomes. As a complement to these efforts, iVirus 2.0 focused on core virus-targeted datasets with high level of curation and ecological context for iVirus-powered analyses. To this end, we mined the literature to capture and aggregate smaller, relevant virus datasets and made them available to download via the CyVerse Data Store. This grew available “virus” data ~20-fold (from 5.5 to 109 billion reads) to now over 10 TB of sequencing data (see Supp. Table 2). These datasets exist as “flat files”, downloadable via CyVerse’s HTTPS endpoint, as well as the Discovery Environment, with the former requiring no logins. Included are sequencing data from global ocean sampling, increasingly diverse soil datasets, the human lung and gut, as well as specialty environments like glacial ice, hypersaline brines, and hydrothermal vents. Additionally, where possible, both raw datasets and curated data products are made available, and many datasets have a static, unchangeable, permanent identifier—a DOI—that can be used for data citation with or without a publication (though DOIs are not required). We provide guidance on how anyone can contribute their own data, detailed below.

To maximize iVirus apps and data resource accessibility, we also provide extensive and regularly updated documentation via “live protocols”-powered community resources at protocols.io. For iVirus 2.0, we updated our five original protocols and added three more to now provide documentation for an end-to-end virus ecogenomics pipeline and development stage updates. The “live protocol” capabilities maximize user feedback, which with such an active user community quickly identifies issues and needs. Further, the protocols.io shared community experience is invaluable for establishing consensus and best practices in a nascent field such as virus ecogenomics. Thus, we have a built-in software development life cycle that helps iVirus best serve the research community. Lastly, yearly hands-on international viromics meetings university-specific microbiome informatics courses provided a critical venue for gathering user feedback, comments and suggestions.

Finally, we revamped our website (https://ivirus.us) as yet another means to disseminate information on protocols, workshops, research, apps and data resources. Complementing this, we maintain technical documentation at bitbucket (https://bitbucket.org/MAVERICLab/ivirus), and have established Singularity and/or Docker containers for all iVirus apps, which allows research labs to run analyses on their own resources, independent of either cyberinfrastructure if preferred.

## Limitations and future opportunities

Though we have sought to develop iVirus through an extensive community-engaged design cycle with specific capabilities for (dsDNA) virus researchers, there are other options available for analyzing virus sequencing datasets. For example, for researchers that have already identified virus contigs in their dataset, stand-alone or web-based platforms exist for analysis (summarized comparative feature sets in Table 3). From these tools one could assess and visualize gene content and sequence variation across populations using tools such as Anvi’o [64], or leverage key comparative genomic and contextualization features available from microbiome-centric platforms including Mgnify [65] and IMG/VR [23]. As described above, IMG/VR is notable for providing a curated set of virus sequences that has been taxonomically classified and assessed for quality, and users can submit their sequences for standardized annotation and analysis. Across all these platforms, iVirus is unique in that it provided virus-centric tools and a modular set of apps across multiple platforms, where users control the specifics of their processing pipeline. This allows flexibility in the tools each user wants to use as the field advances, and to facilitate community awareness of the best practices, benchmarking, and consensus guidelines described above [27, 50, 66].

**Table 3:** Comparative overview between platforms to analyze virome data.

*Category*	*Capability*	*CyVerse*	*Kbase*	*MGnify*	*IMG/VR*
data	Private upload	x	x	x	x
	Public release	x	x	x	x
	Search and integrate in analysis	x	x	x	x
Virus apps	Can add new apps	x	x		
	Cloud-based only?	x	x	x	x
Virome analysis	Contig annotation/AMGs	x	x		x
	Virus detection	x	x		
	Population genetics	x			
	Taxonomy	x	x		

While iVirus 2.0 provides unique and state-of-the-art capabilities for virus ecogenomics analyses, it does carry some limitations. First, virtually all the analytics are for dsDNA viruses; future work will benefit from integrating ssDNA- and RNA- virus-specific analytics and workflows, such as those accumulating through efforts by the European Virus Bioinformatic Center (http://evbc.uni-jena.de/). Second, iVirus data repositories are currently “flat files” that will benefit from more sophisticated focus on best database management system practices to better serve these data and automate data acquisition from decentralized data repositories. Finally, as apps, data, and platforms grow, documentation and app integration, management and maintenance become challenging, which will undoubtedly require a distributed co-laboratory community effort moving forward [67].

Though work remains to feed the appetites of a growing virus ecogenomics community, iVirus 2.0 offers choice in platform (KBase and CyVerse cyberinfrastructures or Singularity containers for local or private cluster set-up), a centralized and modernized set of virus ecogenomic apps and data resources, and mechanisms for usage and community feedback (documentation via “live protocols” at protocols.io and workshops). Collectively, we hope that these efforts will empower dsDNA, and eventually all, virus research across diverse systems.

## Supplementary information

Supplementary Table 1

Supplementary Table 2

## References

[1] Hall EK, Bernhardt ES, Bier RL, Bradford MA, Boot CM, Cotner JB (2018). Understanding how microbiomes influence the systems they inhabit. Nature Microbiol.

[2] Gilbert JA, Blaser MJ, Caporaso JG, Jansson JK, Lynch SV, Knight R. Current understanding of the human microbiome. *Nat. Med.* 2018;24:392–400. 10.1038/nm.4517 PMC7043356 29634682

[3] Suttle CA (2007). Marine viruses-major players in the global ecosystem. Nat Rev Microbiol.

[4] Zimmerman AE, Howard-Varona C, Needham DM, John SG, Worden AZ, Sullivan MB, et al. Metabolic and biogeochemical consequences of viral infection in aquatic ecosystems. *Nat Rev Microbiol.* 2020;18:21–34. 10.1038/s41579-019-0270-x 31690825

[5] Howard-Varona C, Lindback MM, Bastien GE, Solonenko N, Zayed AA, Jang HB (2020). Phage-specific metabolic reprogramming of virocells. ISME J.

[6] Sullivan MB, Lindell D, Lee JA, Thompson LR, Bielawski JP, Chisholm SW (2006). Prevalence and evolution of core photosystem II genes in marine cyanobacterial viruses and their hosts. PLoS Biol.

[7] Lindell D, Jaffe JD, Johnson ZI, Church GM, Chisholm SW (2005). Photosynthesis genes in marine viruses yield proteins during host infection. Nature.

[8] Hurwitz BL, Hallam SJ, Sullivan MB (2013). Metabolic reprogramming by viruses in the sunlit and dark ocean. Genome Biol.

[9] Thompson LR, Zeng Q, Kelly L, Huang KH, Singer AU, Stubbe J (2011). Phage auxiliary metabolic genes and the redirection of cyanobacterial host carbon metabolism. Proc Natl Acad Sci.

[10] Gazitúa MC, Vik DR, Roux S, Gregory AC, Bolduc B, Widner B (2021). Potential virus-mediated nitrogen cycling in oxygen-depleted oceanic waters. ISME J.

[11] Vik D, Gazitúa MC, Sun CL, Zayed AA, Aldunate M, Mulholland MR (2021). Genome-resolved viral ecology in a marine oxygen minimum zone. Environ Microbiol.

[12] Rosenwasser S, Ziv C, Creveld SG, van, Vardi A (2016). Virocell metabolism: metabolic innovations during host–virus interactions in the ocean. Trends Microbiol.

[13] Emerson JB, Roux S, Brum JR, Bolduc B, Woodcroft BJ, Jang HB (2018). Host-linked soil viral ecology along a permafrost thaw gradient. Nat Microbiol.

[14] Trubl G, Jang HB, Roux S, Emerson JB, Solonenko N, Vik DR (2018). Soil viruses are underexplored players in ecosystem carbon processing. mSystems.

[15] Zhong Z-P, Tian F, Roux S, Gazitúa MC, Solonenko NE, Li Y-F (2021). Glacier ice archives nearly 15,000-year-old microbes and phages. Microbiome.

[16] Zhong Z-P, Rapp JZ, Wainaina JM, Solonenko NE, Maughan H, Carpenter SD, et al. Viral ecogenomics of arctic cryopeg brine and sea ice. *mSystems.* 2020;5:e00246–20. 10.1128/mSystems.00246-20 PMC7300359 32546670

[17] Anantharaman K, Duhaime MB, Breier JA, Wendt KA, Toner BM, Dick GJ (2014). Sulfur oxidation genes in diverse deep-sea viruses. Science.

[18] Gao S-M, Schippers A, Chen N, Yuan Y, Zhang M-M, Li Q (2020). Depth-related variability in viral communities in highly stratified sulfidic mine tailings. Microbiome.

[19] Correa AMS, Howard-Varona C, Coy SR, Buchan A, Sullivan MB, Weitz JS (2021). Revisiting the rules of life for viruses of microorganisms. Nat Rev Microbiol.

[20] Blazanin M, Turner PE. Community context matters for bacteria-phage ecology and evolution. *ISME J.* 2021;1–10. 10.1038/s41396-021-01012-x PMC8528888 34127803

[21] Gregory AC, Zayed AA, Conceição-Neto N, Temperton B, Bolduc B, Alberti A (2019). Marine DNA viral macro- and microdiversity from pole to pole. Cell.

[22] Gregory AC, Zablocki O, Zayed AA, Howell A, Bolduc B, Sullivan MB (2020). The gut virome database reveals age-dependent patterns of virome diversity in the human gut. Cell Host and Microbe.

[23] Roux S, Páez-Espino D, Chen IA, Palaniappan K, Ratner A, Chu K, et al. IMG/VR v3: an integrated ecological and evolutionary framework for interrogating genomes of uncultivated viruses. *Nucleic Acids Res.* 2021;49:1–12. 10.1093/nar/gkaa946 PMC7778971 33137183

[24] Nayfach S, Páez-Espino D, Call L, Low SJ, Sberro H, Ivanova NN (2021). Metagenomic compendium of 189,680 DNA viruses from the human gut microbiome. Nat Microbiol.

[25] Roux S, Matthijnssens J, Dutilh BE. Metagenomics in virology. *Encycloped Virol*. 2021;133–40. Published online 2021 Mar 1. 10.1016/B978-0-12-809633-8.20957-6.

[26] Warwick-Dugdale J, Solonenko N, Moore K, Chittick L, Gregory AC, Allen MJ (2019). Long-read viral metagenomics captures abundant and microdiverse viral populations and their niche-defining genomic islands. PeerJ.

[27] Roux S, Solonenko NE, Dang VT, Poulos BT, Schwenck SM, Goldsmith DB (2016). Towards quantitative viromics for both double-stranded and single-stranded DNA viruses. PeerJ.

[28] Simmonds P, Adams MJ, Benkő M, Breitbart M, Brister JR, Carstens EB (2017). Consensus statement: Virus taxonomy in the age of metagenomics. Nat Rev Microbiol.

[29] Roux S, Adriaenssens EM, Dutilh BE, Koonin EV, Kropinski AM, Krupovic M (2018). Minimum Information about an Uncultivated Virus Genome (MIUViG): a community consensus on standards and best practices for describing genome sequences from uncultivated viruses. Nat Biotechnol.

[30] Bin Jang H, Bolduc B, Zablocki O, Kuhn JH, Roux S, Adriaenssens EM (2019). Taxonomic assignment of uncultivated prokaryotic virus genomes is enabled by gene-sharing networks. Nat Biotechnol.

[31] Nishimura Y, Yoshida T, Kuronishi M, Uehara H, Ogata H, Goto S. ViPTree: the viral proteomic tree server. *Bioinformatics.* 2017;33:2379–80. 10.1093/bioinformatics/btx157 28379287

[32] Moraru C, Varsani A, Kropinski AM (2020). VIRIDIC-a novel tool to calculate the intergenomic similarities of prokaryote-infecting. Viruses.

[33] Pons JC, Paez-Espino D, Riera G, Ivanova N, Kyrpides NC, Llabrés M. VPF-Class: taxonomic assignment and host prediction of uncultivated viruses based on viral protein families. *Bioinformatics.* 2021;37:1805–13. 10.1093/bioinformatics/btab026 PMC8830756 33471063

[34] Bolduc B, Youens-Clark K, Roux S, Hurwitz BL, Sullivan MB (2017). iVirus: facilitating new insights in viral ecology with software and community data sets imbedded in a cyberinfrastructure. ISME J.

[35] Merchant N, Lyons E, Goff S, Vaughn M, Ware D, Micklos D (2016). The iPlant Collaborative: cyberinfrastructure for enabling data to discovery for the life sciences. PLOS Biol.

[36] Teytelman L, Stoliartchouk A, Kindler L, Hurwitz BL (2016). Protocols.io: virtual communities for protocol development and discussion. PLOS Biol.

[37] Kindler L, Stoliartchouk A, Gomez C, Thornton J, Teytelman L, Hurwitz BL. VERVENet: the viral ecology research and virtual exchange network. *PeerJ.* 2021; in press.

[38] Arndt D, Grant JR, Marcu A, Sajed T, Pon A, Liang Y (2016). PHASTER: a better, faster version of the PHAST phage search tool. Nucleic Acids Res.

[39] Sousa AL de, Maués D, Lobato A, Franco EF, Pinheiro K, Araújo F, et al. PhageWeb—web interface for rapid identification and characterization of prophages in bacterial genomes. *Front Genet*. 2018; 9. 10.3389/fgene.2018.00644 PMC6305541 30619469

[40] Tynecki P, Guziński A, Kazimierczak J, Jadczuk M, Dastych J, Onisko A. PhageAI—bacteriophage life cycle recognition with machine learning and natural language processing. bioRxiv 2020; 2020.07.11.198606.

[41] Wommack KE, Bhavsar J, Polson SW, Chen J, Dumas M, Srinivasiah S (2012). VIROME: a standard operating procedure for analysis of viral metagenome sequences. Standards Genom Sci.

[42] Roux S, Faubladier M, Mahul A, Paulhe N, Bernard A, Debroas D (2011). Metavir: a web server dedicated to virome analysis. Bioinformatics.

[43] Arkin AP, Cottingham RW, Henry CS, Harris NL, Stevens RL, Maslov S (2018). KBase: The United States department of energy systems biology knowledgebase. Nat Biotechnol.

[44] Roux S, Enault F, Hurwitz BL, Sullivan MB (2015). VirSorter: mining viral signal from microbial genomic data. PeerJ.

[45] Bolduc B, Jang HB, Doulcier G, You Z-QZ, Roux S, Sullivan MB (2017). vConTACT: an iVirus tool to classify double-stranded DNA viruses that infect Archaea and Bacteria. PeerJ.

[46] Hurwitz BL, Westveld AH, Brum JR, Sullivan MB (2014). Modeling ecological drivers in marine viral communities using comparative metagenomics and network analyses. Proc Natl Acad Sci.

[47] Guo J, Bolduc B, Zayed AA, Varsani A, Dominguez-Huerta G, Delmont TO (2021). VirSorter2: a multi-classifier, expert-guided approach to detect diverse DNA and RNA viruses. Microbiome.

[48] Ren J, Kai S, Chao D, Nathan A, Ahlgren, JA, Fuhrman, YL, et al. Identifying viruses from metagenomic data using deep learning. Quant Biol. 2020;8:64–77. 10.1007/s40484-019-0187-4. 10.1007/s40484-019-0187-4 PMC8172088 34084563

[49] Amgarten D, Braga LPP, da Silva AM, Setubal JC (2018). MARVEL, a tool for prediction of bacteriophage sequences in metagenomic bins. Front Genet.

[50] Pratama A, Bolduc B, Zayed AA, Zhong Z-P, Guo J, Vik DR, et al. Expanding standards in viromics: in silico evaluation of dsDNA viral genome identification, classification, and auxiliary metabolic gene curation. *PeerJ.* 2021; In Press. 10.7717/peerj.11447 PMC8210812 34178438

[51] Kieft, K., Zhou, Z. & Anantharaman, K. VIBRANT: automated recovery, annotation and curation of microbial viruses, and evaluation of viral community function from genomic sequences. Microbiome. 2020;8:90. 10.1186/s40168-020-00867-0. 10.1186/s40168-020-00867-0 PMC7288430 32522236

[52] Karner MB, DeLong EF, Karl DM (2001). Archaeal dominance in the mesopelagic zone of the Pacific Ocean. Nature.

[53] Vik DR, Roux S, Brum JR, Bolduc B, Emerson JB, Padilla CCC (2017). Putative archaeal viruses from the mesopelagic ocean. PeerJ.

[54] Vik D, Bolduc B, Roux S, Krupovic M, Sullivan MB. MArVDv2: a machine learning approach to metagenomic archaeal virus detection. bioRxiv 2021; In Press..

[55] Tisza MJ, Pastrana DV, Welch NL, Stewart B, Peretti A, Starrett GJ (2020). Discovery of several thousand highly diverse circular DNA viruses. eLife.

[56] Tisza MJ, Belford AK, Domínguez-Huerta G, Bolduc B, Buck CB (2021). Cenote-Taker 2 democratizes virus discovery and sequence annotation. Virus Evolut.

[57] Shaffer M, Borton MA, McGivern BB, Zayed AA, La Rosa SL, Solden LM (2020). DRAM for distilling microbial metabolism to automate the curation of microbiome function. Nucleic Acids Res.

[58] Edwards RA, McNair K, Faust K, Raes J, Dutilh BE (2016). Computational approaches to predict bacteriophage–host relationships. FEMS Microbiol Rev.

[59] Galiez C, Siebert M, Enault F, Vincent J, Söding J. WIsH: who is the host? Predicting prokaryotic hosts from metagenomic phage contigs. *Bioinformatics*. 2017;33:3113–14. 10.1093/bioinformatics/btx383 PMC5870724 28957499

[60] Nayfach S, Camargo AP, Schulz F, Eloe-Fadrosh E, Roux S & Kyrpides NC. CheckV assesses the quality and completeness of metagenome-assembled viral genomes. Nat Biotechnol 2021;39:578–85. 10.1038/s41587-020-00774-7. 10.1038/s41587-020-00774-7 PMC8116208 33349699

[61] Gregory AC, Solonenko SA, Ignacio-Espinoza JC, LaButti K, Copeland A, Sudek S (2016). Genomic differentiation among wild cyanophages despite widespread horizontal gene transfer. BMC Genom.

[62] Brum JR, Sullivan MB (2015). Rising to the challenge: accelerated pace of discovery transforms marine virology. Nat Rev Microbiol.

[63] Gregory AC, Gerhardt K, Zhong Z-P, Bolduc B, Temperton B, Konstantinidis KT, et al. MetaPop: a pipeline for macro- and micro-diversity analyses and visualization of microbial and viral metagenome-derived populations. *bioRxiv* 2020; 2020.11.01.363960. 10.1186/s40168-022-01231-0 PMC8922842 35287721

[64] Eren AM, Esen ÖC, Quince C, Vineis JH, Morrison HG, Sogin ML (2015). Anvi’o: an advanced analysis and visualization platform for ‘omics data. PeerJ.

[65] Mitchell AL, Almeida A, Beracochea M, Boland M, Burgin J, Cochrane G (2019). MGnify: the microbiome analysis resource in 2020. Nucleic Acids Res.

[66] Solonenko SA, Ignacio-Espinoza JC, Alberti A, Cruaud C, Hallam S, Konstantinidis K (2013). Sequencing platform and library preparation choices impact viral metagenomes. BMC Genom.

[67] Wood-Charlson EM, Anubhav, Auberry D, Blanco H, Borkum MI, Corilo YE (2020). The National Microbiome Data Collaborative: enabling microbiome science. Nat Rev Microbiol.

