# Supplementary material for: iVirus 2.0: Cyberinfrastructure-supported tools and data to power DNA virus ecology
Source: ISME Commun. 2021 Dec 14;1:77. doi: 10.1038/s43705-021-00083-3 (PMC9723767; doi:10.1038/s43705-021-00083-3)
Supplement: Supplementary file 1 — Supplementary Table 1 [file 43705_2021_83_MOESM1_ESM.docx]

**SOM Table 1: Non-exhaustive list of major toolsets and analytical platforms to study microbes. Active as of June 2021.**

| **Category** | **Name** | **Reference** | **Link to resource** |
| --- | --- | --- | --- |
| Tool/Toolset | GTDB | Parks et al., 2018 | [link](https://gtdb.ecogenomic.org/) |
|  | QIIME 2 | Bolyen et al., 2019 | [link](https://qiime2.org/) |
|  | PEMA | Zafeiropoulos et al., 2020 | [link](https://github.com/hariszaf/pema) |
|  | PUMAA | Mitchell et al., 2020 | [link](https://github.com/keithgmitchell/PUMA) |
|  | anvi’o | Eren et al., 2021 | [link](https://merenlab.org/software/anvio/) |
|  | Australian Centre for Ecogenomics | - | [link](https://github.com/Ecogenomics) |
|  | The Huttenhower Lab | - | [link](https://huttenhower.sph.harvard.edu/tools/) |
| Platform | MG-RAST | Meyer et al., 2008 | [link](https://www.mg-rast.org/) |
|  | IMG/M | Markowitz et al., 2015 | [link](https://img.jgi.doe.gov/) |
|  | KBase | Arkin et al., 2018 | [link](https://www.kbase.us/) |
|  | Galaxy | Afgan et al., 2018 | [link](https://usegalaxy.org/) |
|  | iMicrobe | Youens-Clark et al., 2019 | [link](https://www.imicrobe.us/) |
|  | Planet Microbe | Ponsero et al., 2020 | [link](https://www.planetmicrobe.org/) |
|  | EBI-MGnify | Mitchell et al., 2020 | [link](https://www.ebi.ac.uk/metagenomics/) |

Separate literature from main text:

1.Mitchell, K. et al. PUMAA: A Platform for Accessible Microbiome Analysis in the Undergraduate Classroom. Frontiers in Microbiology 11, 1–15 (2020).

2.Bolyen, E. et al. Reproducible, interactive, scalable and extensible microbiome data science using QIIME 2. Nature Biotechnology 37, 852–857 (2019).

3.Afgan, E. et al. The Galaxy platform for accessible, reproducible and collaborative biomedical analyses: 2016 update. Nucleic acids research 44, W3–W10 (2016).

4.Zafeiropoulos, H. et al. PEMA: A flexible Pipeline for Environmental DNA Metabarcoding Analysis of the 16S/18S ribosomal RNA, ITS, and COI marker genes. GigaScience 9, 1–12 (2020).

5.Markowitz, V. M. et al. Ten years of maintaining and expanding a Microbial Genome and metagenome analysis system. Trends in Microbiology vol. 23 730–741 (2015).

6.Meyer, F. et al. The metagenomics RAST server – a public resource for the automatic phylogenetic and functional analysis of metagenomes. BMC Bioinformatics 9, 386 (2008).

7.Parks, D. H. et al. A standardized bacterial taxonomy based on genome phylogeny substantially revises the tree of life. Nature Biotechnology 36, 996 (2018).

8.Youens-Clark, K. et al. iMicrobe: Tools and data-driven discovery platform for the microbiome sciences. GigaScience 8, 1–12 (2019).

9.Ponsero, A. J. et al. Planet Microbe: a platform for marine microbiology to discover and analyze interconnected ‘omics and environmental data. Nucleic Acids Research 1–11 (2020) doi:10.1093/nar/gkaa637.

10.Eren, A. M. et al. Anvi’o: an advanced analysis and visualization platform for ‘omics data. PeerJ 3, e1319 (2015).

11. Arkin, A., Cottingham, R., Henry, C. et al. KBase: The United States Department of Energy Systems Biology Knowledgebase. Nat Biotechnol 36, 566–569 (2018).

12. Mitchell, Alex L., et al. MGnify: the microbiome analysis resource in 2020. Nucleic acids research 48.D1: D570-D578 (2020).
