# Supplementary material for: iVirus 2.0: Cyberinfrastructure-supported tools and data to power DNA virus ecology
Source: ISME Commun. 2021 Dec 14;1:77. doi: 10.1038/s43705-021-00083-3 (PMC9723767; doi:10.1038/s43705-021-00083-3)
Supplement: Supplementary file 2 — Supplementary Table 2 [file 43705_2021_83_MOESM2_ESM.docx]

**SOM Table 2: iVirus-powered datasets on CyVerse**

| **Name** | **Reads (Base pair)** | **Reads (Number)** | **Contigs** | **Viral contigs** | **Viral populations** | **Reference** |
| --- | --- | --- | --- | --- | --- | --- |
| Gut Virome Database (GVD) | 5,350,000,000,000 |  |  | 57,605 | 33,242 | Gregory, A. C. *et al.* 2020. |
| ABOR | 207,400,956,765 | 899,961,906 |  |  |  | Gregory, A. C. *et al.* 2021 |
| Barrow Viromes 2018/2019 | 534,021,784 | 62,627,559,970 | 1,382,682 |  | 1318, 1305 | Zhong, Z.-P. *et al.* 2020 |
| Howard-Varona_2016_ISMEJ_transcriptomics |  |  |  |  |  | Howard-Varona, C. *et al.* 2016 |
| Howard-Varona_2018_ISMEJ_transcriptomics_and_proteomics |  |  |  |  |  | Howard-Varona, C. et al. 2018 |
| Du_SCI_2021 | 63,800,769,354 | 353,217,112 | 3,635,728 | 2,675 | 1,028 | Du, J. et al. 2021 |
| DNA Viromes library comparison | 171,929,283,714 |  |  |  |  | Roux, S. *et al.* 2016 |
| Freshwater Virophage |  |  | N/A | 29 | 31 | Roux, S. *et al.* 2017 |
| Global Ocean Virome 2 (GOV2) | 3,922,769,300,000 | 38,839,300,000 |  | 848,507 | 488,130 | Gregory, A. C. *et al.* 2019 |
| Gazitua/Vik ETSP Viruses | 210,045,456,112 | 1,405,731,534 |  |  | 46,127 | Vik, D. *et al.* 2020 |
| Howard-Varona/Vik Phage | 60,000,000 | 100,000 | 2 | 2 | 2 | Howard-Varona, C. *et al.* 2018 |
| Lung Virome | 19,471,189,500 | 77,884,758 |  |  |  | Gregory, A. C., Sullivan, M. B., Segal, L. N. & Keller, B. C. 2018 |
| Pseudoalteromonas Omics |  |  |  |  |  | Howard-Varona, C. *et al.* 2020 |
| TOV 43 | 437,369,491,202 | 4,330,391,002 | 3,821,756 | 6,322 | 5,476 | Brum, J. R. *et al.* 2015 |
| Tibet Glacier 2017 | 12,612,230,172 | 140,567,847 | 1,849 | 43 | 33 | Zhong, Z.-P. *et al.* *bioRxiv* 2020 |
| Trubl Soil Viromes | 60,600,000,000 | 600,000,000 |  |  | 66 | Trubl, G. *et al.* 2019 |
| Vik 2017 | 931,665,782 |  | 49,022 | 344 | 43 | Vik, D. R. *et al.* 2017 |
| efam |  |  | 242,078 |  |  | Zayed, A.A., *et al.* 2021 |
| **BPs** | 10,457,524,364,385 | 109274714129 | 5,069,283 | 915,527 | 574,178 |  |
| **GBps** | 10457.52436 | 109.2747141 |  |  |  |  |
